# Socio-ecological trajectories in a rural Austrian region from 1961 to 2011: comparing the theories of Malthus and Boserup via systemic-dynamic modelling

**DOI:** 10.1080/1747423X.2020.1820593

**Published:** 2020-09-27

**Authors:** Claudine Egger, Helmut Haberl, Karl-Heinz Erb, Veronika Gaube

**Affiliations:** Institute of Social Ecology, University of Natural Resources and Life Sciences, Vienna, Austria

**Keywords:** System-dynamics, agricultural intensification, LTSER, population pressure, land-use change, technological progress

## Abstract

This paper investigates to what extent the theories of Thomas Robert Malthus and Ester Boserup are still useful to analyse population and land-use trajectories in an industrial society at a regional scale. Following a model-based approach toward long-term socio-ecological research, we built two system dynamic models, each representing one theory, and calculated socio-ecological trajectories from 1961 to 2011 for a study region located within the Eisenwurzen region in Austria. Comparing the model trajectories with empirical data reveals opposing results for the fit of the dynamics of ‘population and technology’ compared to ‘land use and technology’. Technology strongly influenced population development, whereas its impact on land-use intensity faded over time. Although these theories are usually seen as opposing, both models identify population development as a main driver for land-use changes, mainly population decreases that contributed to farmland abandonment. We find out-migration to be essential when applying the investigated theories to contemporary societies.

## Introduction

A growing population and decreasing available agricultural land per capita motivate inquiries into the relations between population and the availability and use of land. Among other factors, these relations are mediated by technology (Grübler & Messner, 1998; Haberl et al., 2016). The interplay between population dynamics, technological change and land-use change is key to understanding socio-ecological trajectories. The most prominent, and antagonistic, theorists who related population, land and technology were Thomas Robert Malthus and Ester Boserup. The frameworks of both theorists were not intended to be applied to industrial societies. For industrial societies a vast body of literature exists that examines the complexity of the various drivers of changes in agricultural land use (Bürgi et al., 2005; Hersperger & Bürgi, 2009; Mottet et al., 2006; Van Vliet et al., 2015). Van Vliet et al. (2015) provide an excellent overview of more than 130 case studies in Europe, showing that economic, technological, institutional and location factors have often been identified as underlying drivers, while demographic and socio-cultural factors have been less frequently mentioned. In this context, the purpose of this paper is not to add to this literature by analysing observed land-use changes in a case study through that lens. Instead, we aim to understand the extent to which the Boserupian and Malthusian theories are still applicable in an industrial society by singling out the population-land-use relationship, acknowledging that more factors need to be included to get a more ‘realistic’ representation.

It is difficult to overestimate the extent to which Tomas Robert Malthus’ famous book *An Essay on the principles of population*, first published in 1798, influenced the debate on the interrelations between the dynamics of human populations and the land on which they depend. Under the impression of the rapid population increase in the late 18^th^ century, Malthus argued that population growth was exponential (he called it ‘geometrical’) and hence bound to outgrow the growth of resource provision, which he assumed to be linear (he called it ‘arithmetical’). His main claim was that the resulting mismatch between the growth of population and that of resources would inevitably lead to what he called ‘positive checks’, i.e. crises like famines, wars, emigration, etc. that would reduce population size to a sustainable level. In Malthus’ theory, technology is assumed to be an independent, external factor that only affects land, specifically its productivity (Malthus, 1992).

Ester Boserup is widely acknowledged as Malthus’ most prominent antagonist (Fischer-Kowalski et al., 2014). In influential books such as Boserup (1965) and Boserup (1981), Boserup developed concepts for how population size, more exactly population density, and land use and in this context agricultural technologies influence each other, thereby contradicting Malthusian dismal prophecies. Based on a cultural-anthropological approach, she studied the relations between the dynamics of population, technology and land use for different societies (hunter and gatherer, agrarian) and concluded that population (density) drives the adoption of technology and in consequence land use (land demand), even at high social costs such as a decline in labour efficiency and increased workloads (Boserup, 1981, 1965). In later years, Boserup formalized these hypotheses in her development framework, describing changing relations of the key variables for the transition from hunter-gatherer to industrial societies (Boserup, 1996).

The work of Boserup and Malthus is mostly discussed in the context of attempts to analyse land-use transitions in agrarian societies as well as societies in early phases of industrialization. Studies based on their theories often concentrate on the population-land relation and the impact of population pressure on land use (Demont et al., 2007; Desiere & D’ Haese, 2015; Malmberg & Tegenu, 2007; Meertens et al., 1996; Rasmussen et al., 2012; Stéphenne & Lambin, 2001). These theories have rarely been applied in studies of industrial societies, however. This gap motivated us to focus on trajectories within a developed industrial society (Austria) and ask, what can be learned today from the propositions of Boserup and Malthus when analysing regional development in industrial societies over decadal time spans.

Industrial societies are characterized by a low agrarian labour force, abundant energy supply required for high grades of mechanization, a high division of labour and high per-capita incomes (Gingrich & Krausmann, 2018; Krausmann, 2004). In Austria, as in most Western European countries, the economic take-off with fast technological changes affecting the economic sectors as well as the societies themselves happened after the Second World War. This development theoretically aligns with Boserup’s proposition of ‘induced agricultural intensification’. Interestingly, the period after the Second World War also coincides with the renaissance of Malthusian philosophy in Western societies. The publication of books like ‘Road to Survival’ (Vogt, 1948), ‘The Population Bomb’ (Ehrlich, 1968), and ‘Limits to Growth’ (Meadows et al., 1972) fostered neo-Malthusian narratives that are centred around overpopulation and its detrimental consequences. Population growth has since been stylized as essential cause of resource depletion, environmental degradation, and significant contributor to climate change, as critically analysed by Hendrixson and Hartmann (2019).

For this study, we chose a model-based approach to long-term socio-ecological research (LTSER). This approach is often followed in anthropology and archaeology to study development trajectories of ancient populations, e.g. Anasazi, Easter Island (Axtell et al., 2002; DiNapoli et al., 2020; Lee & Tuljapurkar, 2008), whereas we here use it to analyse a rather recent period. We developed two simple system-dynamic models, one Boserupian (the B-model) and the other Malthusian (the M-model) and applied both to a regional case study in Austria for the period 1961–2011.

Many models stemming from the thinking of Boserup and Malthus undertake a transition analysis between Malthusian and Boserupian development phases and their roles in land system change with theoretical models in artificial simulation environments (Decker & Reuveny, 2005; Magliocca et al., 2013; Puleston et al., 2014). Our goal was to build simple models that are applicable to the case study region and allow the comparison of the simulation trajectories with empirical data. We did not aim to comprehensively explain or even forecast socio-ecological dynamics, but rather to compare two prominent theories claiming to be able to explain population–land-use relationships, in an attempt to understand the extent to which they still hold today, and which main additional factors are required to match contemporary trajectories. We exploit the ability of system dynamic (SD) models to analyse dynamic relations in complex systems with indirect feedback loops (Rasmussen et al., 2012; Stéphenne & Lambin, 2001; Verburg, 2006) between our key variables technological progress, population and land use.

Our main focus is on the analysis of the relations between population and land use, and we study how technological progress has affected them. To what extent can simple models mimic the observed socio-ecological trajectories in that region? Can they still help representing these dynamics for a regional system? As neither Malthus’ nor Boserup’s theories were intended to be applied to industrialized societies, we aim to identify factors they do not consider but are crucial for the understanding of socio-ecological processes in industrial societies.

## Methodology

### Model region

**Figure 1.**
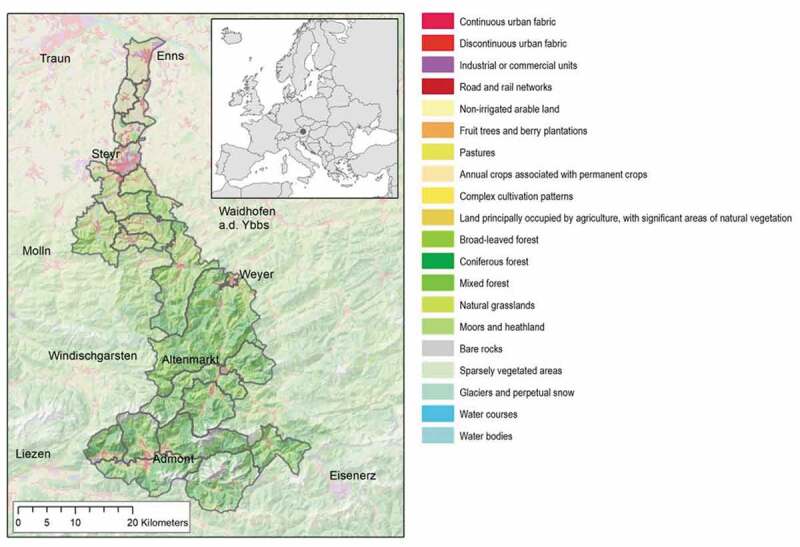
Depicts the Enns valley case study region with its urban centre the city of Steyr, and main land-use distinctions. The inset map illustrates the location of the study region in Austria and Europe. Own drawing, Source: (‘Basemap – Verwaltungsgrundkarte von Österreich,’ 2018)

The study region is a part of the so-called Eisenwurzen region, which is an established Long-Term Socio-Ecological Research (LTSER) platform (Peterseil et al., 2013). Situated along the Enns valley in Upper Austria and Styria, it includes 21 municipalities (see Figure 1). Its area totals 1,271 square kilometres (km^2^). The average altitude of the municipalities varies between 310 and 658 meters above sea level, with highest points reaching about 2000 meters above sea level (BEV, 2015). Along the study region the climate zones range from Central European climate of transition with mean annual temperatures from 9°C and precipitation rates of 750 mm in the north, to alpine climate with 4°C and about 2000 mm in the south.

Two characteristics make this area an interesting study site. First, the transect covers a large fraction of the diversity of Austrian agriculture and reflects a broad variety of central European farming landscapes. The lowlands in the area around and north of Steyr are characterized by industrialized agriculture (mostly cropland mixed with intensive livestock rearing), high population density and urban centres with industrial and commercial activities. With rising altitudes towards the south east, population density and farming intensity go down and agriculture is primarily extensive livestock rearing (grazing). In the south, the importance of forestry is also higher.

Second, the area has undergone contrasting forms of development. It is a sub-territory of the so-called Eisenwurzen (literally ‘origin of the iron’), a region around the Erzberg (literally ‘ore mountain’) with a long-standing tradition of mining and iron industry. In the middle ages, the area experienced prosperous socio-economic development due to its large iron ore deposits. Resource scarcity (mainly wood scarcity) forced labour division already in early times and resulted in intense trading relations by interchanging food for manufactured products with the neighbouring regions. The decline of the iron industry at the end of the 19th century induced an agricultural retransformation accompanied by high emigration rates. Today the region is part of the LTSER (long time socio ecological research) platform and site for many studies of society-nature interaction (Hasenauer et al., 2007) such as studies on future regional development (Gaube et al., 2009), historical analyses of energetic shifts in the agroecosystem (Gingrich et al., 2018), denitrification potential of land-use (Schroeck et al., 2019) or legacy effects of land-use systems on carbon stocks (Thom et al., 2018).

### Model structure

In land-use science, modelling is a cost-efficient approach that allows applying, testing and modifying different socio-ecological concepts for study regions of varying scales. Especially when historical land systems are studied, models facilitate exploring the role of potential drivers in affecting past land management practices and altering land-use systems.

For social-ecological modelling, Verburg et al. (2016) distinguish five generic model categories ranging from deterministic biophysical and economic, reduced complexity, agent-based and simple models. Deterministic models such as earth system or general equilibrium models are not useful in our context, as they require large data and computing power and due to their low capability of integrating feedbacks (Verburg et al., 2016). To study the external, structural influences land-use models can be applied on the individual level by analysing impacts on decision-making of single agents (e.g. farms) via agent-based modelling (Padró et al., 2019) or on an aggregate level focusing on long-term effects and systemic mechanisms of specific land-use systems (Sylvester et al., 2015).

Given the goal of our study, we hence chose a reduced-complexity social-ecological model. System dynamic models are often used for scenario comparisons and model implementation of theories that build on top down system definition and feedback loops. Our modelling approach allows us to simulate complex dynamics with relatively simple assumptions, as we see our models primarily as learning tools (Brown et al., 2013; Verburg et al., 2016)

**Figure 2.**
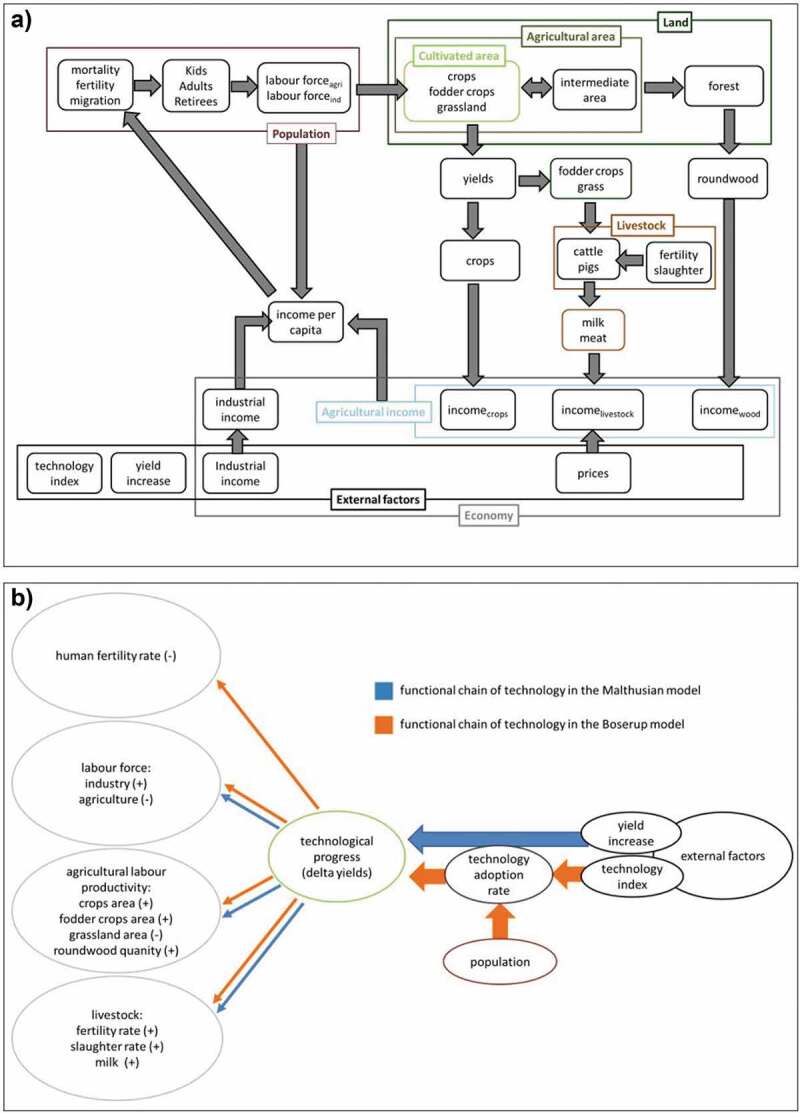
(a) Shows the common structural elements of both models. (b) Depicts the model specific origins of technology as well as its functional relations (indicated by the positive or negative signs) with the model variables listed in the bubbles

Our models share the same fundamental architecture, which is built upon the five components population, land, technology, livestock and the economy. According to the diverging concepts of Boserup and the Malthus, they only differ by their assumptions regarding the origin of technological progress and its feedbacks. The main intention is to study systemic relations through comparison of their different model projections with real-world trajectories of the study region. Hereafter, we first describe the common structure and then highlight the key differences between the M-model and the B-model regarding population and technology. Table 1 and Figure 2 depict the main structure and characteristics of the two models.

**Table 1.** Description of the structure and dynamics for the B-model and the M-model

	M-Model	B-Model
Dynamics	technology -> land -> population	population -> technology -> land
Population	total population = kids _age<15_ + adults _age 15–65_ + retirees _age > 65_	
	population dynamics: – scenario LE: migration rate depends negatively on income – scenario SC: migration rate depends positively on sectoral labour migration (decrease of agricultural labour force)	
	division of labour is influenced by technological progress -> allowing for migration from agriculture to industry	
Land	land: cultivated area + intermediate area + forestcultivated area: cropland + fodder cropland + grasslandintermediate area: temporarily uncultivated area, that can be recultivated in the next yearforest: can not be (re-)transformed into agricultural area	
	land-use dynamics: –cultivatable area per farmer is either limited by its initial value augmented by tech. progress or by the amount of available agricultural land–technological increase forces crops and fodder crops cultivation and reduces grassland–intermediate area: 1/30 in each year is lost due to transition into forest	
Technology	external factor	internal factor
	– technological increase: avg. harvest increase over all field types (t/ha/yr) – harvest increase: based on the 50 years avg. yearly increase for each field type (fixed amount)	– tech. increase: avg. harvest increase (t/ha/yr) over all field types – technology index: ratio of the non-agricultural income to the agricultural income as proxy for technological growth – population density: positively affects the technology adoption rate – harvest increase: the grade of the adopted technology then impacts the harvest increase
Animals	cattle and pig stocks are influenced by slaughtering and fertility rates, which are bound to fodder cops and grassland	
Economy	open economy: long lasting trading relation of the region influenced the decision to build an open economy model–all prices and industrial income are external factors–agricultural income in each year newly computed

#### Common model structure

Total **population** development depends on the interplay between the demographic factors ageing, reproduction, mortality, and migration. Therefore, total population is divided into three cohorts: children below the age of 15, retirees above the age of 65 and adults in between. In both models, the gender distribution is assumed as being equal, with females having a reproductive cycle of 30 years (Statistics Austria, 2019; World Health Organization, 2006). The initial age and mortality distribution were derived from national-level data of Statistics Austria and applied to the model populations (Statistics Austria, 2016a, 2015). This rather peripheral and rural area is characterized by out-migration that decreases the adult population, which we model based on two different mechanisms in the scenarios LE (‘low earnings’) and SC (‘structural change’) explained below. The adults represent the total labour force that enters a two-sector economy of the model, consisting of agriculture and industry. While the income of workers in the industrial sector is a given external factor, the agricultural income has to be earned in every period by selling crops, milk, animal meat (beef and pork) and wood. Technological progress affects the initial labour rate division by forcing labour migration from agriculture to industry.

To study **land** transition dynamics, the total land area was fixed to the initial value 127,070 ha (Statistics Austria, 2014c). Land area is subdivided into agricultural area (separated into crops, fodder crops, grassland and fallow) and forest. Only changes from agricultural area to forests are allowed because the Austrian forestry legislation forbids (re-)transformation of forest into agricultural area (Gesamte Rechtsvorschrift für Forstgesetz, 1975). The agricultural labour force is used in three land-use types, crops, fodder crops and grassland, which sum up to the cultivated area. As the cultivated area changes in each year, the temporarily uncultivated land is subsumed in the intermediate area, which can be re-cultivated but is gradually transitioning into forest. Assuming the prevalence of natural succession, and to take probabilistic changes into account, in each year, 1/30 of the intermediate area transforms into forest and then cannot be re-cultivated (Baur & Binder, 2015). The cultivable land per farmer is limited either by the amount of available agricultural land per farmer or by its initial value augmented by technological progress to account for higher productivity through technological improvements.

The entire crop harvest is sold, whereas fodder crops and grassland yields are used as animal feed. Technological progress acts in a labour-saving manner and decreases the share of the agricultural labour force. Further, as total land is fixed, it forces land-use change via preference change, as a higher technology index stimulates the preference for crop production and vice versa reduces the cultivation of grassland.

The forest is used for wood production. The round wood production per worker is derived from FAO statistics and its productivity is increases with technological progress.

With respect to **livestock**, only cattle and pig stocks are considered. Cattle and pig stocks are influenced by fertility and slaughtering rates. The animal stock sizes, respectively the animal fertility is assumed to be bound to the available fodder crop land (pigs) and grassland (cattle). These functions reflect the legal restrictions on animal stocking density (Gesamte Rechtsvorschrift für Wasserrechtsgesetz, 1959) and guarantee fodder sustenance. It is assumed that half of the cattle stock provides milk and half meat. The total milk production is influenced by the ratio of grass (fodder) to fodder crops and positively influenced by technology.

The **economy** is subdivided into two sectors, agriculture and industry. The industrial sector represents a combination of manufacturing and services. The history of the area, with its long-lasting trade relations, has influenced the decision to build an open economy model. Therefore, production (agricultural and non-agricultural) is assumed to be traded at the world market and all prices as well as the industrial income are external factors.

#### M-model

Along with Malthusian theory, in the M-model higher income leads to decreases of mortality and increases of fertility and hence to population growth. The core of Malthusian theory is the assumption that technological progress is exogenous and results in a linear increase in yields (Malthus, 1992). Thus, yields are increased in the M model by a fixed amount in each year. This value was set based on the long-term (50 years period) average crop yield growth of each crop type.

#### B-model

In her later, more comprehensive framework, Boserup describes how evolving technology alters family structures through organizational and cultural changes, reflecting that better technology helps to raise the education and formal employment of women and thereby affects the fertility rate (Boserup, 1996, 1981). This is reflected in the model through a two-parted fertility rate, counterbalancing the positive relation of income and fertility by an additional, negative feedback loop of technology on fertility.

The internal representation of technological progress in the B-model also results from yield increases but with a different mechanism compared to the M-model (see Figure 2(b)). Based on the ratio of the agricultural and the non-agricultural income, we created a technology index as a general proxy for technological progress in Austria. In a Boserupian notion, higher population density positively influences the adoption (% rate) of technological progress from the technology index, which then results in yield increases. To develop the operationalization of technology for the B-model was one of the most difficult model settings. As discussed in Box 1, it was not possible to simply model technology via an easily available proxy variable such as fertilizer consumption. Further, as it was unrealistic to assume that technological innovations are generated in the model region, it was necessary to choose a non-regional approach and base the technology index on national variables.

#### Two scenarios for out-migration

Since population development is the linchpin of both theories, the models simulate two different population scenarios. The scenarios have a diverging population development, resulting from different assumed causes of out-migration. Both migration scenarios depend on model dynamics. In the ‘low earning’ scenario LE, emigration depends negatively on income, implying that low incomes in the region stimulate out-migration while rising incomes lead do declining rates. The ‘structural change’ scenario SC is driven by structural changes in the labour sectors, meaning higher shifts from the agricultural to the industrial sector lead to higher movement to industrial centres outside of the study region.

### Data and assumptions

For building the models, national data were used to derive functional relationships between individual components of the models, e.g. on technology and yield increases, in order to avoid regional biases – except the region-specific migration rate for the ‘structural change’ scenario, which was calculated with regional data from Statistics Austria. The national data were mostly derived from the Statistics Database of the Food and Agriculture Organization (FAOstat), but due to missing data tables for specific indicators, they were enhanced by World Bank and Statistics Austria datasets. The models are initialized and validated with regional data on population, livestock and land-use data described in Table 2Table 3.

**Table 2.** Summary of the used data and main assumptions for model construction, (Food and Agricultural Organization, 2016a, 2016c, 2016d, 2016e, 2016f; Statistics Austria, 2014a, 2014b, 2014c, 2014d, 2015, 2016b; Worldbank, 2016)

Data for computation			
Data	Source	Time series	Comments
land: crops, fodder crops, grassland (area, yield), roundwood productionanimals: animal stocks (pig and cattle), meat production (pig and cattle), milk production	FAOstat	1961–2011	assumption: all land types are mapped to the three categories crops, fodder crops and grassland
agricultural prices: crop prices	FAOstat	1966–2011	in local currency assumption: of constant prices from 1961–66weighted yields and prices according to the % area share for representative products
demographics: fertility rate, mortality rate	World bank	1961–2011	assumption: to fit both model theories, the fertility rate is splitted into an increasing and a decreasing function
economics: gdp components (industrial income, agricultural income)	World bank	1974–2011	in local currency interpolated data for the missing yearsratio of non-agricultural to agricultural income
agricultural labour force	Statistics Austria	1960,1970,1974–2011	interpolated data for the missing years
emigration rate (regional)		1961–2011	10 years delta
population		1961–2011	intial value from 1961
labour force		1970–2010	interpolated value for 1960
animal stocks (pig and cattle)		1960–2010	interpolated value for 1960 as average of 1950 and 1970
agricultural areas		1959–2010	initial value from 1959

**Table 3.** Comparison of the model results

Results	M-Model	B-Model	Fig.
Population			
Total Population			
1960s – 1980s	LE: both models predict a decline of population, underestimate empirical dataSC: both models predict a linearly increasing population, slightly overestimate empircal data		**3A**
1980s – 2000s	population reaches a tipping point,model population trajectories start to drift apart		
	LE and SC: strong population development, linear population increases	LE: stagnating population, coincides with empiric dataSC: linear population increase, followed by flattening development	
2000–2010s	population overshoot in both scenariosLE: estimated population in the end + 37% SC: estimated population in the end +112%	LE: population trajectory aligns with empirc population, in the end −1%SC: decreasing population trajectory, overall overestimation of +36%	
Population Structure			
1960s – 1980s	uniform model predictions: children and retirees remain stable in both scenarios, difference manifested in the adults group LE: decreasing in the first decade, recovery in the second SC: stable increase		**3B and 3 C**
1980s – 2000s	parallel development of retirees for both scenarios and models, divergent model paths for adults and children		
	LE: continuing, linear increase for adults and childrenSC: same development with steeper curves and higher population levels	LE: flattening curve for adults towards the end of the period, children peak mid period and decrease from then onwardsSC: same development on higher population levels	
2000–2010s	LE and SC: linear increases for all population groups	LE and SC: increases for the retirees, adults stagnate,decreases for children sizes, number of children end in both scenarios at the same niveau	
Agriculture			
Forest			
1960s – 1980s	LE and SC: both models show similar, increasing trajectories, but different slopes for each scenario, both models underestimate the increase of forest shares but reach emirical data towards the end of the periode		**3 F**
1980s – 2000s	deviation of the models trajectories in the second half,model dynamics start to dominate the scenario effects		
	LE: increasing model curve corresponds to the real data pointsSC: overall, notable misfit strongly underestimates forest development	LE: increasing model curve shows a very close fit to empric dataSC: underestimates forest development, convergence with empric towards the end	
2000–2010s	LE and SC: continuing linear increase, with flattening curves, SC on lower niveau	LE and SC: steep increases, both curves end almoust at the same, high niveau	
Cropland			
1960s – 1980s	LE and SC: both models show similar, increasing trajectories, but different slopes depending on the scenario		**3D**
1980s – 2000s	LE and SC: deviation of the models trajectories (earlier in E2), model dynamics start to dominate the scenario effects		
	LE: underestimates the copland development SC: overestimates the copland development	LE: underestimates the copland development SC: aligns with the oscillations of the empric data	
2000–2010s	LE: converges towards empric dataSC: curve overshoots	LE and SC: show a structural break in the last period and converge in both scenarios towards 3–4% of cropland shares	
Grassland			
1960s – 1980s	both models show similar trajectories but with different slopes for each scenario LE: model curves show notable misfit by overestimating drops of grassland shares SC: the trajectories match about the decreasing development of the empirical data, especially the B-Model		**3E**
1980s – 2000s	deviation of the models trajectories in the second half, model dynamics start to dominate the scenario effects		
	LE and SC: linarly decreasing curves on different niveaus depending on scenario, SC curve is close to emprical data	LE: strong deviation from empiric data and notable misfitSC: similar development with empiric data, strong deviations depicting major drops of grassland shares	
2000–2010s	slightly decreasing curves on different niveausLE: underestimates grassland sharesSC: shows a good fit to empric data	LE and SC: convergence of both models, both curves overestimate the decrease of grassland (5–10%)	

The model is built upon linear regressions on the yield differences Δx from the base year 1961 (t = 0) and the given year t, Δx being

$$\Delta x=\frac{x_{t}-x_{t=0}}{x_{t=0}}$$

As both theories explicitly mention retrogressions, the deviation from the initial value is computed for each year, which allows to model forward (g(∆x)>0) and backward (g(∆x)<0) development.

$$f\left(x_{t}\right)=y_{t}=x_{t=0}*\left(1+g\left(\Delta x\right)\right)$$

The modelling period is set from 1961 to 2011; this 50-year time frame was considered long enough to observe several business cycles as well as generational effects on population.

The model structure is built upon three major assumptions. First the varying land-use classes were mapped to the field types: crops, fodder crops and grassland. Second, it was necessary to create reference crop types and prices, for which four crop cultures, respectively three for grassland, were chosen and weighted by their relative area shares. These weights were used to compute yields and prices. Third, as in many other industrial countries (Herzer et al., 2012), the Austrian fertility rate is contradicting Malthusian theory as it increases with GDP per capita until the mid-1960s, from then on decreases, respectively stagnates for the later decades. Based on the idea for a fertility function from Prskawetz et al. (1999), the actual fertility rate was split into an increasing and a decreasing function to fit for both model structures. For the first term, resembling the Malthusian notion of fertility, a simple linear increasing function was constructed by interpolating a series for the first two observations. The second function is calculated as the difference between the Malthusian and the actual fertility rate – the first two elements interpolated vice versa. Since Boserup perceived fertility as influenced by positive and negative impacts, the B-model fertility rate inherits both terms.

## Results

**Figure 3.**
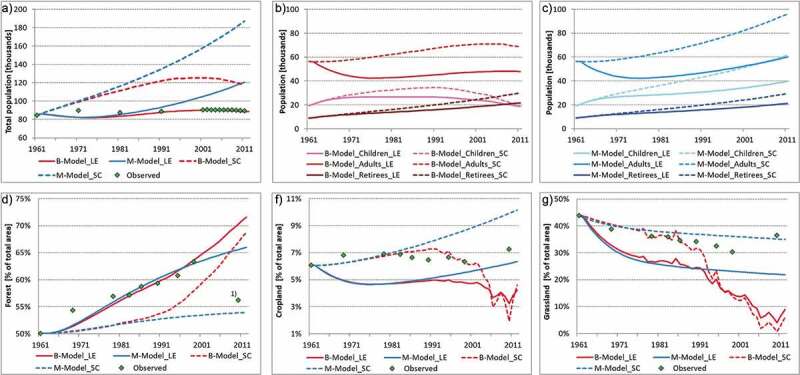
Scenario-model trajectories for population development and land-use dynamics of the B-model and the M-model for the two scenarios ‘low-earning’ (LE) and ‘structural change’ (SC): (a) compares the total population development and highlights the negative impact of migration and technology (B-models) on population growth. (b-c) depict changes in population structure (children, adults and retirees) and show the negative impact of technology on fertility by the decline of kids and raise of retirees (B-models in B) in contrast to linear population development (M-models in C). (d-f) depict the development of the relative shares [%] of forest, cropland and grassland of the two models. Empirical data show fast acceleration of land use change (first two decades), followed by stabilization with oscillations (contradicting technology development). Further the broad variation of scenario trajectories show the impact of different population sizes (M-models) and the impact of technology (B-models last two decades). 1) Due to a restructuration of the forest administration law there were major changes in the allocation of forest areas to municipalities; this last data point was hence found to be incomparable with the others and excluded from the analysis

Figure 3 shows the modelled trajectories for population and land-use development in comparison with empirical data. Figure 3(a) depicts the pathways for the total population, while B and C highlight details on the population structure. Figure 3(e-f) illustrate the development for Cropland, Grassland and Forest shares.

The observation period is divided into three intervals 1960–80, 1980–2000 and 2000–2010. The period 1960–2010 is characterized by three major changes in the agro-political structure that must be taken into consideration for the analysis and comparison of the model trajectories with the empirical data. Since the end of the Second World War until 1980, the Austrian government set emphasis on production increases and self-sufficiency. From the 1980s onwards until 2000, Austria’s preparation for the entry into the European Union and upcoming environmental programs encouraged extensive farming practices. The last decade from 2000 until 2010 is already influenced by the EU policy Agenda 2000 that bundled individual agricultural programmes into the common EU policy (CAP) (Kammer & Rohrmoser, 2012; Kröger, 2006).

For 1960–1980, the empirical data show a steady increase of population in the first decade followed by stabilization, but an overall population growth of 3% until 1980. In either scenario, the B-model and the M-model present uniform development. Both models fail to adequately reflect the observed population development. In LE the models predict an initial decline of population, which is contrasted by linear population growth in SC. Figure 3(b and c) give insights into the underlying changes in the population structure of the models. While the number of children and retirees remain stable, the difference between the scenarios manifests in the number of adults. In LE their number is strongly decreasing in the first decade with average out-migration rates of 5% (M-model) and 3% (B-model), contrary to a stable increase in SC.

In 1961 forest and grassland shares clearly dominate land use (95%), complemented by minor shares of cropland (crops and fodder crops subsumed). The empirical data show high increases for forest and cropland shares in the first period, whereas grassland shares notably decrease. For all three land-use categories, both models again show similar trajectories, but depending on the scenario different slopes. For forest, either scenario underestimates the sharp rise in forest shares, but the LE-trajectory approximates the historic values towards the end of the modelled period. For cropland and grassland, LE depicts major declines, while SC predicts small deviations and corresponds relatively well to the empirical development.

In the period 1980–2000, the observed population development is characterized by stagnation and total population shows only a minor increase. At the beginning, the modelled populations reach a tipping-point at which the trajectories for the M- and the B-model start to develop differently. The M-model shows sustained linear growth and overestimates the population development in both scenarios. In contrast, the B-models projects flattening population curves and catches the observed trend. While the SC curve overestimates population growth, the stagnating trajectory in scenario LE aligns well with empirical population development. Figure 3(b and c) again show a parallel development of retired persons for scenarios and models, while the other model paths differ greatly. In both population trajectories LE and SC, the M-model predicts continuous linear growth of the adult and child groups. In contrast, the B-models predict flattening curves for adults towards the end and the curves for children reach their peak in the middle of the period and decrease from then on.

The empirical land-use data show an unstable development. While forest cover continues to increase, shares of arable land and grassland shift up and down. As seen with population, the model paths for land use start diverging in the second half of the period. For forest development, the LE trajectories of both models correspond well to the observed data points, while SC starts far off, but the B-model trajectory converges towards the empirical data. In contrast, for cropland and grassland the SC paths of both models match the empirical data whereas the LE curves do not fit.

In the last period (2000–2010), the empirical population data are characterized by stagnation with minor oscillations around 90.000 inhabitants. The population development now rather aligns along the model groups than the scenario. The M-models overshoots the population for 2011 considerably in either scenario, by +37% in LE and in SC even by +112%. In contrast, the curve for LE in the B-model depicts a remarkably good fit with empiric population development and misses the last observation only by −1%. In SC, the B-model overestimates also the population size for 2011 by +36%.

Looking at the dynamics of population groups in Figure 3(b and c), the M-models still show linear increases for all groups, while the B-models depict an increase for the group of retirees, stagnation among adults and a decline in the number of children. The empirical data depict a trend reversal for land use, as forest shares drop to about 55%, whereas cropland and grassland shares increase to 7% and 37%.

The linear curves of the M-models, LE for cropland, respectively SC for grassland match closest to the empiric data observations in the last period. The B-model trajectories converge towards each other and strongly deviate from the M-model. Despite the close fit of the B-model trajectories of SC before with cropland and grassland development, the B-model curves show the biggest deviation to the empirical data with projections of cropland shares of 3–4% and grassland decreases to 5–10%.

## Discussion

From our analysis of the population scenarios, we conclude that technology has a strong impact on population. In our comparison, the better performance of the B-Models for population development generally confirms the Boserupian idea of technology influencing the family structure (Boserup, 1996). As Figure 3(b and c) show, the main difference for population between the models originates in the diverse development of children, as the B-models depict the negative impact of technology on fertility and correctly predict aging of society as the number of children falls below that of retirees.

Both models fail to adequately grasp the land-technology nexus for the studied land systems, hence indicating that models capturing the role of technology need to go beyond both classical approaches when analysing contemporary industrialized societies (Krausmann et al., 2008; Lemmen, 2011; Turner & Fischer-Kowalski, 2010). Boserup starts off along with Malthus, reflecting the population-land relation, in her development framework. Later, she replaces this connection through a technology-land link for industrial societies (Boserup, 1996). The increasing misfit of the B-model trajectories the overall fit of the M-model trajectories, with constant technological progress, and the comparison of the empirical land-use data with the agricultural index (used as proxy for technology) indicate a declining impact of technological progress on land in the study region. Furthermore, the strong differences of the model paths between the two scenarios emphasize the human impact on land-use dynamics. This leads us to two questions: First, why do our land-use data not reflect technological progress? And second, what is the impact of population on land-use dynamics?

In contrast to a simplified model, in the real world, technology describes a complex multi-level and multi-factor system that cannot be captured by one-factor explanations. There is a broad field of research on the adoption and diffusion of agricultural technologies highlighting individual traits (Schewe & Stuart, 2015), social networks (Rogers, 2003), learning (Sassenrath et al., 2008) or farm structure (Napier & Tucker, 2001), as well as the production factors labour and capital (Levins & Cochrane, 1996). While our representation of technology worked for the analysis of population for an industrial society, it seems important to be more specific in the case of agricultural technology. No uniform effect of technology seems plausible, the respective context and the socio-technical relationship should be taken into account (Bingham, 1996; Schewe & Stuart, 2015). Technological progress can stimulate farm growth and more intense production. On the other side, it can result in the opposite effect as farms convert to part-time farming, when the ‘gained’ labour time is substituted by off-farm work (Weiss, 1999).

Besides technology, institutional drivers and location factors can have ambiguous impacts and stipulate intensification as well as de-intensification processes (Van Vliet et al., 2015). In a comparable study on landscape change for a Swiss study region in the period of 1930–2000, Hersperger and Bürgi (2009) draw similar conclusions from their findings: low importance of technology on land-use processes on a local level, which they explain by the initially already high development level and the location of the study site. In contrast, political and economic aspects are the dominant drivers identified in their analysis (Hersperger & Bürgi, 2009). Turner and Ali (1996) identified for a study in Bangladesh for the period 1950–1986 the institutional and environmental constraints as one key influences for agricultural stagnations. The geographical location of our study site in the alpine landscape in Austria conditions an environment for small-scale dairy farming and hereby limit possible advancement in size and efficiency gains through technology.

With limited options for technological advancement the participation of farms in agri-environmental programs can be important, and hence their instruments or measures have a strong influence on farm management practices (Hambrusch et al., 2006). From our scenario runs we see that governmental policies and land use are closely linked and the latter mirrors the effects from national and international governmental interventions. In the early post-war period, the agricultural sector had a key role in securing food supply and in releasing labour force for industrial production. Agricultural productivity was actively forced through substitution of labour by machines (mechanisation), increase of fertilizer application and improved seeds; leading to a drop of the agricultural workforce and intensification and modernisation of the agricultural production (Kammer & Rohrmoser, 2012; Kröger, 2006). The fall in prices that resulted from the ensuing overproduction, social dissatisfaction with the resulting changes in agriculture, and preparations for EU accession in 1994 then led to realignment of the agricultural programs in the 1980s. The new subsidies program (ÖPUL) focussed on sustainable production with socio-ecological balance, quality products and increasing organic production (BMLFUW.gv.at, 2017; Kammer & Rohrmoser, 2012). Followed by the reform of the Common Agricultural Policy (CAP) and the Agenda 2000, with a combined approach for agriculture and transregional development (Kammer & Rohrmoser, 2012).

Besides policies and geographic limitations, an important factor that has often been neglected as driver of land-use change is culture and its impact on shaping the cultural landscape (Bürgi et al., 2005). Cultural traditions can have a strong influence on the long-term relationship of societies and their land management, as historical practices can have a persistent influence on prevailing land-use techniques (Mottet et al., 2006).

This leads to our second question regarding the implications of both models for the impact of population on land-use dynamics. The changing population structure (overaging) of the B-models depict a demographic transition, which is highly influential for agricultural changes, since retirement and succession impact long term investments in and survival of farms (Van Vliet et al., 2015).

The second aspect, the influence of migration on land use, is illustrated in particular by the differences in the development paths of the M-models. Neither Boserup nor Malthus has specific explanations for the causes of migration, although the diverse population development trajectories of our models stress its long-lasting, socio-ecological effects. Continued migration of adults can lead to the collapse and abandonment of settlement areas (McLeman, 2011) and migration plays a crucial role of agricultural transitions in industrial countries. Depopulation of rural areas is one of the major concerns in the European Union (Collantes et al., 2014; Pašakarnis et al., 2013; Van Berkel & Verburg, 2011). The economically driven migration rates of the LE scenario are in stark contrast to the empirical data of our study region, which suggests that non-economic, structural effects may be more important in motivating out-migration. Other research on rural-urban migration in Austria stress the effects of distance to economic centres and housing prices (Helbich & Görgl, 2010), local public infrastructure especially for education (Schipfer, 2005) and in a European comparison even gender related social concepts (Leibert & Wiest, 2016) as origins for rural out-migration.

Although, migration has been a main driver for land-use change in Europe since the middle ages (Antrop, 2005), studies mostly either focus on migration or land-use transitions, but generally do not simultaneously consider the social, economic and ecologic dynamics of both of these factors (Chen et al., 2014). Chen et al. (2014) combine land use (Foley et al., 2005) and mobility (Zelinsky, 1971) transition descriptions and illustrate how the transformation from subsistence to intensive agriculture is accompanied by increasing rural mobility and massive migration toward urban centres. In modern societies, migration is only one symptom of extensive mobility. Boserup and Malthus both did not foresee how globalized markets would boost mobility and couple distant socio-economic and environmental actions in modern societies. Taking teleconnections into account that link distinct areas in functional relationships to each other (Friis et al., 2016; Liu et al., 2018; Seto et al., 2012), urbanization processes can have different effects on land systems, as the demand for conservation and recreational land use from urban areas may prevent agricultural intensification in peri-urban areas (Lambin et al., 2001). This goes hand in hand with a change in the social perception of land use with a shift in focus from production towards other benefits of agricultural landscapes such as recreation and ecosystem services (Vejre et al., 2007).

In Austria, agriculture, tourism, and economic output are closely linked. Besides food production, agriculture has an important role in upholding the rural infrastructure in these zones (Kröger, 2006). Our study region contains an urban centre in the northern part (the city of Steyr) and lies embedded within the proximities of the federal capitals Salzburg, Linz and Graz and is also well accessible from Vienna. Furthermore, with the declaration of the two national parks Gesäuse and Kalkalpen and the emphasis on the cultural history of the Eisenwurzen region (iron craftsmanship, agricultural landscapes) the EU structural LEADER programs focused on a tourism-based transregional development strategy for this region (Hasenauer et al., 2007).

Comparing the modelling results with empirical data reveals that both model approaches, despite their fundamental differences, each have specific strengths and can depict certain dynamics relatively well. None of the models performs better than the other in overall terms. This confirms the position by Lee, who sees Boserup and Malthus not so much as opponents, but rather as complementary (Lee, 1986). From our analysis we conclude Boserupian dynamics between population and technology, but also Malthusian stagnation after an initial path of technologization and intensification of land use.

## Conclusions

Despite their fundamental differences, both models have strengths and provide insights into population and land-use dynamics of a rural region in an industrial society. The B-models highlight changes in population structure and hence stress the importance of demographic factors for land-use development. Their population paths corroborate Boserup’s assumption on the relation of population and technology, even under different conditions than foreseen by Boserup. The land use trajectories, with their gradual transition from population to technology domination, highlight the importance of changes in variable relations. The Malthusian model may not deliver specific answers but highlights the human influence on land use and that population based, socio-cultural factors are important drivers of land-use change worthwhile for further investigation. From the comparison of our model trajectories with empiric data and other research, we identify governmental institutions as highly influential for industrial societies. The occurrence of structural breaks, observed in the data, indicate that national and international political programs, global economic trends and geographic limitations have a strong impact on regional development.

The simple system dynamic models presented in this study allow us to derive important lessons for future applications (e.g. in agent-based models). In particular, it shows that out-migration must be a central model element when applying the theories of Boserup and Malthus to peripheral regions in contemporary industrialized societies. Moreover, it is important to acknowledge and diversify the guiding principles for social behaviour (e.g. susceptibility to technological progress or governmental subsidies).

From our take on Boserup and Malthus, we conclude that industrial societies can learn that population and land use are still closely linked. To understand local land-use changes and long-time socio-ecological development, on the one hand it is key to understand the driving forces of population change, especially the causes and push-factors for out-migration. On the other hand, we plea for more interdisciplinary approaches to understand the multi-layered relation between societies and land use and to investigate drivers for land-use dynamics such as culture, whose impact is recognised but not yet sufficiently understood and analysed.

## Figures and Tables

**Table:** 

**Figure 4.**
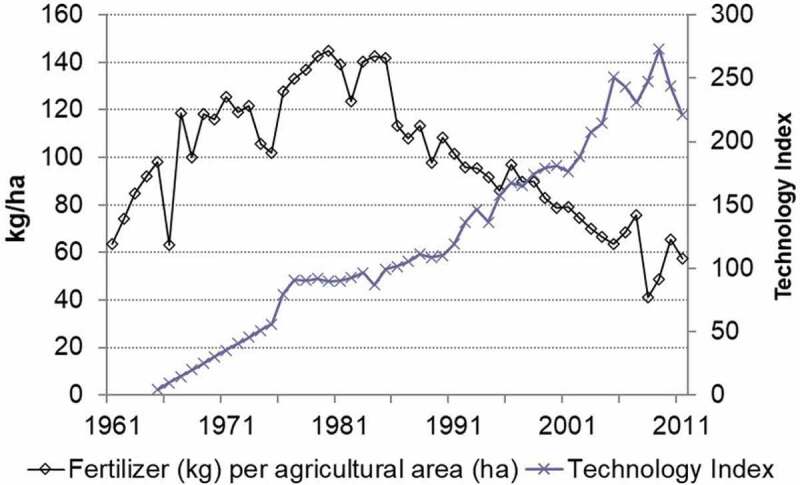
Aggregated consumption of fertilizer (nitrogenous, phosphate, potash, complex fertilizer (K_2_O)) per ha agricultural area contrasted by the Technology Index, Own figure, Source: (EUROSTAT, 2016; Food and Agricultural Organization, 2016b)

***Box 1: Fertilizer as proxy variable for technology?*** In many cases, the consumption of mineral fertilizer is used as an indicator for technological progress (Doss, 2006; Beshir et al., 2012; Khanna, 2001; Lambrecht et al., 2014; Morris et al., 2007). Following this approach, originally the technological progress for the B-Model was designed to be observed by the fertilizer (nitrogen, potash, ash and potassium) consumption in t/ha/yr on agricultural area (Food and Agricultural Organization, 2016b; EUROSTAT, 2016). As Figure 4 depicts, fertilizer consumption reaches its peak in the mid 1980s and shows a steady decrease from then on, resulting in approximately the same fertilizer consumption in 2011 as 1961, while yields continued to grow throughout the period. Comparison of fertilizer use with population density and harvest yields showed no correlation; therefore fertilizer consumption was ruled out as proxy variable for technological progress. The technology index on the other hand differs from the trajectory of fertilizer use for the second half of the observation period with an unabated growth trend until the economic crisis in the late 2000s. The difficulties with the operationalization of technology emphasize the complexity of capturing multidimensional variables like technology and also reinforce the re-evaluation of ideas and models. Further, as there were still yield gains since the 1980s, the contradictory relationship indicates that fertilizer consumption rather seems to reflect the raised efficiency with which nutrients are supplied to plants then the applied technological level. However, the observed technological impact is indirectly shown via this efficiency aspect by the decrease of fertilizer consumption and the simultaneous yield increase.
